# Variability of UK seagrass sediment carbon: Implications for blue carbon estimates and marine conservation management

**DOI:** 10.1371/journal.pone.0204431

**Published:** 2018-09-24

**Authors:** Alix Green, Michael A. Chadwick, Peter J. S. Jones

**Affiliations:** 1 Department of Geography, University College London (UCL), London, United Kingdom; 2 Department of Geography, King’s College London, London, United Kingdom; Università della Calabria, ITALY

## Abstract

Seagrass meadows provide a multitude of ecosystem services, including a capacity to sequester carbon dioxide (CO_2_) within their sediments. Seagrass research in the UK is lacking and there is no published data on sediment carbon (C) within UK seagrass meadows. We sampled 13 *Zostera marina* meadows along the southwest coast of the UK to assess the variability in their sedimentary organic carbon (OC) stocks. The study sites were considered representative of sub-tidal *Z*. *marina* meadows in the UK, spanning a gradient of sheltered to exposed sites, varying in formation, size and density, but found along the same latitudinal gradient. OC stocks (C_stocks_) integrated across 100cm depth profiles were similar among all sites (98.01 ± 2.15 to 140.24 ± 10.27 Mg C ha^-1^), apart from at Drakes Island, which recorded an unusually high C_stock_ (380.07 ± 17.51 Mg C ha^-1^) compared to the rest of the region. The total standing stock of C in the top 100cm of the surveyed seagrass meadows was 66,337 t C, or the equivalent of 10,512 individual UK people’s CO^2^ emissions per year. This figure is particularly significant relative to the seagrass area, which totalled 549.79 ha. Using estimates of seagrass cover throughout the UK and recent UK C trading values we approximate that the monetary value of the UK’s seagrass standing C stock is between £2.6 million and £5.3 million. The C stock of the UK’s seagrass meadows represent one of the largest documented C stocks within Europe and are, therefore, of important ecosystem service value. The research raises questions concerning the reliability of using global or regional data as a proxy for local seagrass C stock estimates and adds to a growing body of literature that is looking to understand the mechanisms of seagrass C storage. When taken with the fact that seagrass meadows are an important habitat for commercially important and endangered species in the UK, along with their declining health and cover, this research supports the need for more robust conservation strategies for UK seagrass habitats.

## Introduction

Seagrass meadows provide a multitude of ecosystem services, including a capacity to sequester CO_2_ within their sediments [1]. Along with mangroves and salt marshes, the organic C absorbed in these coastal ecosystems has been termed ‘blue carbon’ and has generated considerable interest in recent years, in part because preservation and restoration of these habitats can help mitigate climate change [2]. Unfortunately, seagrasses are declining with estimates that at least 49% of UK seagrass coverage has been lost in the last 35 years [3]. This loss not only removes the sequestration potential of these habitats but can also remineralise sedimentary C that has accumilated over time, leads to a reduction of nursery and feeding habitat for commercially important and endangered speices [4], increases sediment and coastal erosion [5]) and reduces coastline nutrient cycling [6–8].

*Zostera marina*, the UK’s dominant seagrass species, is a temperate seagrass found throughout Europe, the USA and the northwest Pacific. Globally eelgrass is declining by approximately 1.4% per year, with large scale declines in some locations, particularly within Europe and east coast USA, due to wasting disease [9]. Much of the evidence of wasting disease in the UK is anecdotal, and with no complete historic inventory of UK seagrass meadows mapping accurate changes over time is challenging at best. Prior to the outbreak of wasting disease in the 1920s eelgrass would have been found in the majority of subtidal mudflats in Britain, which was once considered ‘clothed’ in eelgrass [10]. Following the outbreak of wasting disease, eelgrass was restricted to only the most sheltered sites, such as lagoons, and is now considered nationally scarce [10]. Meadows that do persist are reportedly in a ‘perilous state’, damaged and degraded, and healthy beds are now a rarity [11].

Despite recognition by the EU Water Framework Directive of seagrass as bioindicators for ecosystem health [12] research related to UK seagrass habitats is lacking relative to other regions (e.g., Med and Aus [13]). More specifically, there are no published estimates for the C stored in the UK’s seagrass habitats. This is surprising considering the proliferation of blue C research in recent years, with key papers [12–16] highlighting the vital role seagrasses play in absorbing CO_2_. Occupying less than 0.2% of the ocean floor, seagrass habitats are estimated to be responsible for approximately 10% of the yearly ocean C burial [13,17], a disproportionately large storage potential relative to their global extent [18]. Seagrasses produce aboveground foliage forming canopies in the water column, which slow water, forcing sediment to settle and become trapped within the canopy layer. In this way particles from the water column are absorbed into their sediments, where the overwhelming majority of the C stored by these habitats is located [8]. On average 2.51 ± 0.49 Mg C ha is stored in the living biomass (roots and rhizomes) of seagrass compared to 194.2 ± 20.2 Mg C ha in sediment [13]. This process means that seagrasses can store C through both photosynthesis (autochthonous) and through trapping particles containing C that has come from external sources (allochthonous) such as seston, algae or debris of terrestrial origin. A global assessment of studies suggest that up to 50% of the C stored by seagrass is allochthonous, making seagrasses particularly affective C sequesters since they bind C that could be released back into the ocean by other less stable sinks [8]. Seagrasses form understory mats, made up of dense root systems that stabilise sediments and bind C [17]. These mats can extend to over 10m and create anaerobic sediments that, if left undisturbed, can bind C for millennia [19,20]. In comparison, terrestrial soils whose productivity is often dependent on soil turnover, tend to bind C for decades only [21].

Seagrass ecosystems likely represent a ‘*globally significant carbon stock*’, with estimates suggesting that 19.9 Pg C is stored in the top 1m of the worlds’ seagrass sediments, equivalent to the global fossil fuel and cement production in 2014 [13,22]. The Fourqurean paper [13] has done much to increase awareness and has propelled seagrass into blue C research focus. However, values are derived from regional estimates with between 1 and 29 data points and Mediterranean and Australian habitats comprise 42% of the total data points from this study [13]. Further, the North Atlantic averages are from only 24 samples, none of which are from UK waters [13]. With such limited available data, these studies have been useful in promoting the advancement of seagrass C research. The challenge is that limited data means these estimates are biased regionally, and by species, so tend to generalise storage capture trends [23]. *Posidona* oceanica, a seagrass species found throughout the Mediterranean and known to be exemplar in its ability to store C, dominates the literature, which has been evidenced to skew regional and global extrapolations [23]. Variations in C storage among species, and among habitats formed of the same species, are known [23,24], but the characteristics that affect this, and the impact of habitat distinction are less well understood [23–25]. Direct measurements from regions and species that are under-represented will help to improve global knowledge and develop more reliable estimates of the C storage capacity and potential of seagrasses. For countries where blue C research has developed further there has been a move towards incorporating it into domestic climate policy, going so far as to discuss the inclusion of blue C stocks within Greenhouse Gas (GHG) inventories [26]. Clearly the next step toward successful integration of blue C policy is more robust estimates of C storage across the different blue C habitats.

This study aims to document the C storage in seagrass beds along the southwest coast of the UK. The study objectives were to: (1) compare sediment organic C (OC) of 13 seagrass meadows, on the same latitude and exhibiting varied habitat features; (2) establish the impact of habitat variance on sediment C storage; (3) estimate the average C stock (C_stock_) per unit area to provide a comparison to global and regional data and; (4) estimate the total C stored within each habitat to understand the significance of the UK’s seagrass habitats. The results will (a) provide a baseline assessment of the UK’s seagrass C storage capacity; (b) build on the growing body of literature comparing seagrass C storage locally and globally; and (c) indicate the potential monetary significance of the UK’s blue C storage for this habitat type.

## Materials and methods

**Fig 1:**
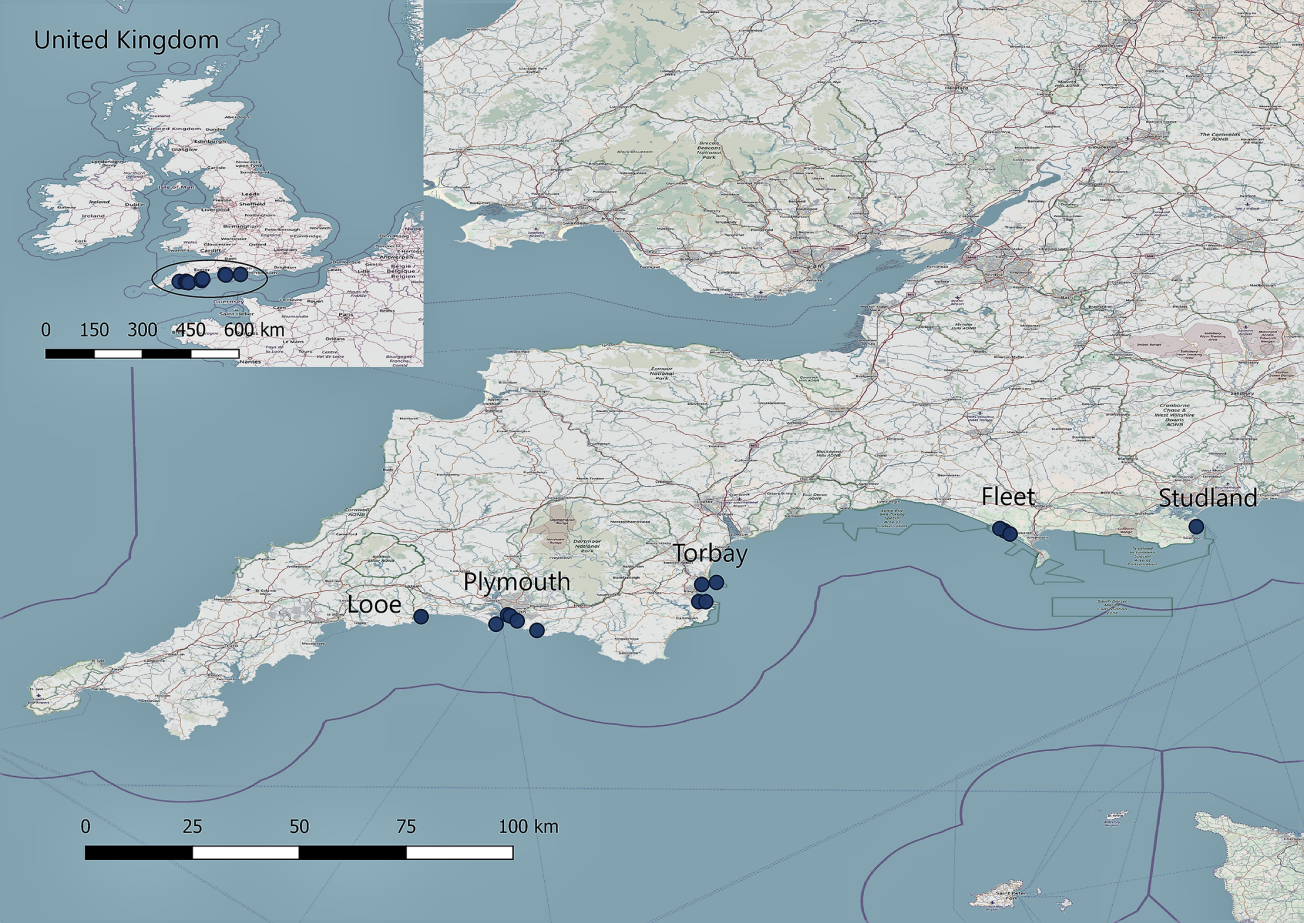
Location of seagrass meadow sites along the southwest coast of the UK.

Thirteen study sites (Fig 1, Table 1) exhibiting varied habitat characteristics were selected for the current study. Sites were considered representative of sub-tidal seagrass meadows found across the British Isles, varying in size, degree of shelter, and formation (Table 1). In addition, sites represented varying degrees of marine protection, ranged from 0.02ha to 275ha and varied in aboveground density. Sites were located on the same latitudinal gradient between 50° 18' 36.36'' and 50° 38' 34.20''N.

**Table 1:** Characteristics of surveyed seagrass meadows.

Site	Protected status	Exposure	Meadow formation	Area (ha)	N	W
Looe	MCZ	Exposed	Very patchy	56.52	50° 21' 11.52''	4° 26' 30.48''
***Plymouth***						
Cawsands	SAC	Partly sheltered	Very patchy	11.77	50° 19' 52.32''	4° 11' 53.52''
Firestone Bay	SAC	Sheltered	Patchy	0.76	50° 21' 37.8''	4° 9' 37.44''
Drakes Island	SAC	Partly sheltered	Dense	4.25	50° 21' 25.56''	4° 9' 10.08''
Jennycliff Bay	SAC	Exposed	Patchy	11.77	50° 20' 27.96'	4° 7' 49.08''
Yealm CC	SAC	Sheltered	Dense	0.14	50° 18' 36.36''	4° 3' 58.68''
Tomb Rock	SAC	Sheltered	Sparse	0.15		
***Torbay***						
Elbery Cove	MCZ	Sheltered	Sparse	29.31	50° 24' 17.64'	3° 32' 41.28''
Torre Abbey	MCZ	Very exposed	Very patchy	104.11	50° 27' 38.52''	3° 32' 1.32''
Fishcombe Cove	MCZ	Very sheltered	Very patchy	0.23	50° 24' 11.52''	3° 31' 17.76''
Hopes Cove	SAC	Partly sheltered	Gradient	2.73	50° 27' 52.56''	3° 29' 16.44''
***Weymouth / Poole***						
Fleet	SAC, SSSI, RAMSAR SPA, UNESCO	Very sheltered	Dense	274.68	50° 37' 72.20''	2° 33' 43.30''
Studland Bay	No protection	Very sheltered	Dense	53.37	50° 38' 34.20''	1° 56' 38.30''

Abbreviations are as follows: MCZ = marine conservation zones, SAC = special area of conservation, SSSI = special scientific site of interest, RAMSAR = convention on wetland of international importance, SPA = special protected area, UNESCO = world heritage. Area values provided by CSI (Community Seagrass Initiative)

Sample collection among all sites were completed in summer 2016 (Fig 1). At each site, three sediment cores were collected from sea depths of 3-8m using SCUBA gear, except at the Fleet, where samples were collected from depths of <0.6m using snorkelling gear. At each site, two divers were dropped from a dive boat, roughly in the centre of the bed, and sampling locations, at least 20m apart, were randomly selected. Permission to collect material was granted by the Marine Management Organisation by providing ‘notice of intention to carry on an activity under The Marine Licensing (Exempted Activities) Order 2011 [27] (as amended) “the Exemptions Order”‘ (EXE/2016/00148). Since the Fleet is property of the Ilchester Estate further permission was provided by the Fleet Warden and by Natural England.

At each location one cylindrical PVC core (70mm diameter, 40cm long) was manually inserted into the sediment to a depth of 30-35cm. Cores were extracted and capped underwater and stored vertically in a lift bag for the remainder of the dive. Once returned to the boat, samples were kept vertically in a covered cool box until arrival on shore. On shore, cores were sliced into 3cm sections, bagged and frozen to await transfer back to the laboratory for analysis. In addition to the sediment cores, three 50cm^2^ quadrats were randomly placed around the core and plant densities were estimated by counting the number of plants within the quadrant. The seagrass meadow at the Fleet was considerably larger than any other bed (Table 1) so three sites were allocated for sampling. The strict protection surrounding the Fleet, and its shallow depth, meant that a kayak was used to reach core locations, so as not to disturb the seagrass. Meadow exposure and bed formation were visually assessed during site visits.

### Laboratory analysis

On returning to the laboratory, samples were thawed and divided into two sub-samples. One sub-sample was used for Loss on Ignition (LOI) analysis and the other was freeze-dried for grain size analysis and total organic C content using an elemental analyser.

#### Organic C and carbonate analysis

Since its presentation in 1974 [28] LOI, the burning of sediments at 550°C and 950°C, has been widely used as a method to estimate the amount of organic matter (OM) and carbonate mineral content in soil samples [29]. The relationship between LOI at 550°C and OM content, and LOI 950°C and carbonate content is accepted as standard [29]. There exists a relationship between OM and OC, which has led to the OM found by burning sediment at 550°C being used as a proxy for OC. However, this method is semi-quantitative and relies on an empirically derived relationship between OC and OM, the strength of which varies with material [30]. The most accurate method to analyse OC is through dry combustion in an Elemental Analyser (EA) [30]. However, the costs involved are often prohibitive. A study analysing the global data set of seagrass C storage demonstrated that the relationship between OM and OC for seagrass sediments is strong, therefore, OM is accepted as a proxy for OC [13,30]. To improve the predictability of OM to OC two linear equations have been developed for samples with %OM higher or lower than 0.2% [13,30]:

%LOI<0.20%OC=‑0.21+0.40(%LOI);

%LOI>0.20%OC=‑0.33+0.43(%LOI).

Although these equations are deemed suitable for OC estimations under IPPC regulations, accuracy can be further improved by sending a limited number of samples to be analysed in an EA [13,30].

The dry mass of each sample was calculated by weighing wet sub-samples before and after drying at 105°C for 18-24hrs [29]. The samples were then put in the furnace at 550°C for two hours, re-weighed and returned for two hours at 950°C [29]. OM content was calculated by subtracting the combusted sediment (550°C) from the sediment dry weight. Carbonate content was calculated by subtracting the remaining combusted sediment (950°C) from the sediment dry weight [29].

Using stratified random sampling 10% of dried samples were selected for analysis in a *Flash EA* (BEIF Lab; UCL, London). Large items, such as roots and shells, were removed by hand before the samples were homogenised. All samples contained significant levels of carbonates so were acidified to remove these before analysis. Sub-samples were submerged in HCL diluted to 1N and placed in an ultrasonic bath for 15 minutes [30]. Samples were then left overnight (>18hours). More acid was added the following day to check for further effervescence and once no new outgassing was observed samples were centrifuged and decanted from the acid. Samples were washed by adding deionised water, sonicated for 15 minutes and then centrifuged to separate the sample and the liquid for decanting. This was repeated three times before the samples were dried at 60°C for >24hrs. The treated samples were then analysed using the *Flash EA* for %C [30].

The %C results represent %OC as the samples had any inorganic C removed prior to analysis. The relationship between %OM (LOI) and %OC was formulated by developing a linear equation for the analysed samples and applying this to the rest of the %OM results.

#### Grain size analysis

Sediment grain size was determined from freeze dried samples from one core for each meadow, which was assumed to be broadly representative of the entire site. Sediment samples were each dry sieved through a sieving tower for 10 minutes. Seven sieves were used; 2mm, 1mm, 0.5mm, 0.25mm, 0.15mm, 0.125mm and 0.054mm. Total mass of sample and mass of retained soil in each sieve was recorded. Sediment silt content was calculated as the percentage of sediment retained below 54μm. Sediment characteristics were further analysed using GRADISTATv8 software [31].

#### Estimating seagrass OC stocks

A C stock (C_stock_) refers to the total amount of C within a habitat of a known size, normally comprising a number of C pools, i.e. reservoirs of C in soil, vegetation etc. [30]. Since the amount of C within the living biomass of seagrass is negligible [13] C_stock_ here refers to the total stock of OC within the sediments of each meadow of a known size.

C_stock_ for each meadow was estimated over a 30cm core sample. Where a 30cm sample was not achieved the missing slices were estimated using the relationships between depth, soil weight from a known volume (dry bulk density, hereafter, DBD) and OC, to determine OC at 3cm intervals up to 30cm [13], <5% of core slices were estimated in this way.

The C_stock_ of the top 30cm of the 13 studied seagrass meadows were calculated as follows. Soil DBD was calculated from the mass of a dried sample and its original volume (*DBD (g/cm*^*3*^) = mass of dry soil (g) / original volume sampled (cm^3^)). Soil C density (SCD) was calculated from DBD and total OC content (SCD = DBD*(OC/100). Total C in each core slice (TC/S) was determined from the SCD and known sample volume (TC/S = SCD*3cm), and, finally, each slice within the core was summed to give total C within a core (*TC/S*_*1*_ *+ TC/S*_*2*_ *+ TC/S*_*n…*_*)*. Values were converted into Mg C/Hectare^-1^ and total C in the top 30cm of each meadow was determined by averaging the total core C and multiplying by area [30]. For global comparisons stock estimates were extrapolated to 100cm following the IPCC protocol [30] and then extrapolated for the whole of the UK to provide estimates of total standing stock. To compare to regional trends units were converted to g C m^2^ and integrated values to 25cm were used.

Statistical comparisons for C_stock_, DBD and plant density were conducted to determine site-specific differences. Test for normality and homogeneity of variance were done to establish if ANOVA or Kruskal Wallace test should be performed. All analysis was completed in SigmPLot 13.0.

## Results

### Seagrass meadow formation and sediment characteristics

The mean DBD in UK seagrass sediments ranged from 0.34 g cm^3^ ± 0.10 (Fleet) to 1.19 g cm^3^ ± 0.09 g cm^3^ (Studland Bay) with an average of 0.96 g cm^3^ ± 0.22 g cm^3^ (Table 2). Mean %OM content ranged from 1.40% ± 0.67% (Studland Bay) to 12.32% ± 5.39% (Drakes Island) with an average of 3.61% ± 3.31% and a median of 2.47%. The Fleet and Drakes Island %OM were markedly higher (Table 2) and differed significantly to all other sites (p <0.001). There was no significant difference between the Fleet and Drakes Island, and no significant difference between all other sites.

**Table 2:** Sediment characteristics and aboveground biomass.

Site	Sediment silt content %	DBD (g cm^3^)	%OM	%OC	SCD (mg C cm^2^)	C_stock_ 30cm (Mg C ha)	Plant density (plants/50cm^2^)
Looe	20.03 ± 1.25	0.98 ± 0.10	2.3 ± 0.76	1.20 ± 0.31	11.08 ± 0.49	33.30 ± 1.47	8.53 ± 6.27
***Plymouth Sound***							
Cawsands	12.72 ± 1.77	1.11 ± 0.12	2.47 ± 0.74	1.25 ± 0.32	14.21 ± 1.08	42.07 ± 3.08	6.08 ± 5.76
Firestone Bay	13.34 ± 2.91	0.86 ± 0.08	3.47 ± 0.55	1.62 ± 0.31	14.19 ± 0.67	40.99 ± 3.38	4.05 ± 5.88
Drakes Island	5.51 ± 1.43	0.77 ± 0.07	12.32 ± 5.39	4.94 ± 2.00	37.76 ± 6.75	114.02 ± 21.45	10.42 ± 8.40
Jennycliff Bay	2.44 ± 0.66	1.07 ± 0.04	2.51 ± 0.43	1.30 ± 0.16	13.89 ± 0.65	39.07 ± 5.35	2.84 ± 4.75
Yealm CC	14.55 ± 1.70	0.87 ± 0.07	2.68 ± 0.48	1.37 ± 0.18	11.83 ± 0.21	35.39 ± 0.70	6.7 ± 7.01
Tomb Rock	8.85 ± 1.29	0.96 ± 0.05	1.85 ± 0.48	1.04 ± 0.21	10.15 ± 0.40	29.40 ± 0.65	4.21 ± 4.51
***Torbay***							
Elbery Cove	21.99 ± 2.46	1.05 ± 0.28	2.59 ± 0.47	1.33 ± 0.18	13.84 ± 0.56	41.74 ± 2.28	10.63 ± 9.45
Torre Abbey	12.02 ± 2.50	1.14 ± 0.09	1.97 ± 0.10	1.10 ± 0.04	12.56 ± 0.50	37.76 ± 1.50	5.52 ± 5.10
Fishcombe Cove	4.81 ± 1.79	1.04 ± 0.10	2.43 ± 0.65	1.28 ± 0.24	13.08 ± 0.72	38.94 ± 2.44	5.71 ± 7.64
Hopes Cove	14.71 ± 1.83	1.09 ± 0.07	1.56 ± 1.84	0.95 ± 0.68	10.73 ± 3.91	30.08 ± 8.89	7.61 ± 5.98
***Weymouth/ Poole***							
Fleet	29.92 ± 5.30	0.34 ± 0.10	9.39 ± 2.95	3.82 ± 1.14	12.07 ± 1.49	37.76 ± 3.84	n.a.
Studland Bay	1.99 ± 0.66	1.19 ± 0.09	1.40 ± 0.67	0.86 ± 0.27	10.13 ± 1.80	37.76 ± 5.39	53.53 ± 10.45

Data are site means ± standard deviation. % silt content; DBD = g dry bulk density; %OM = % organic matter; %OC = % organic carbon; SCD = soil carbon density mg C / cm^2^; C sock Mg C ha = megagrams of C per hectare; plant density = no. plants per 50cm^2^

To determine the relationship between %OM and %OC 10% of samples were analysed in a *Flash EA* (BEIF Lab; UCL, London). A regression analysis determined the relationship as:

%OC=0.3708%LOI+0.3732

**Fig 2:**
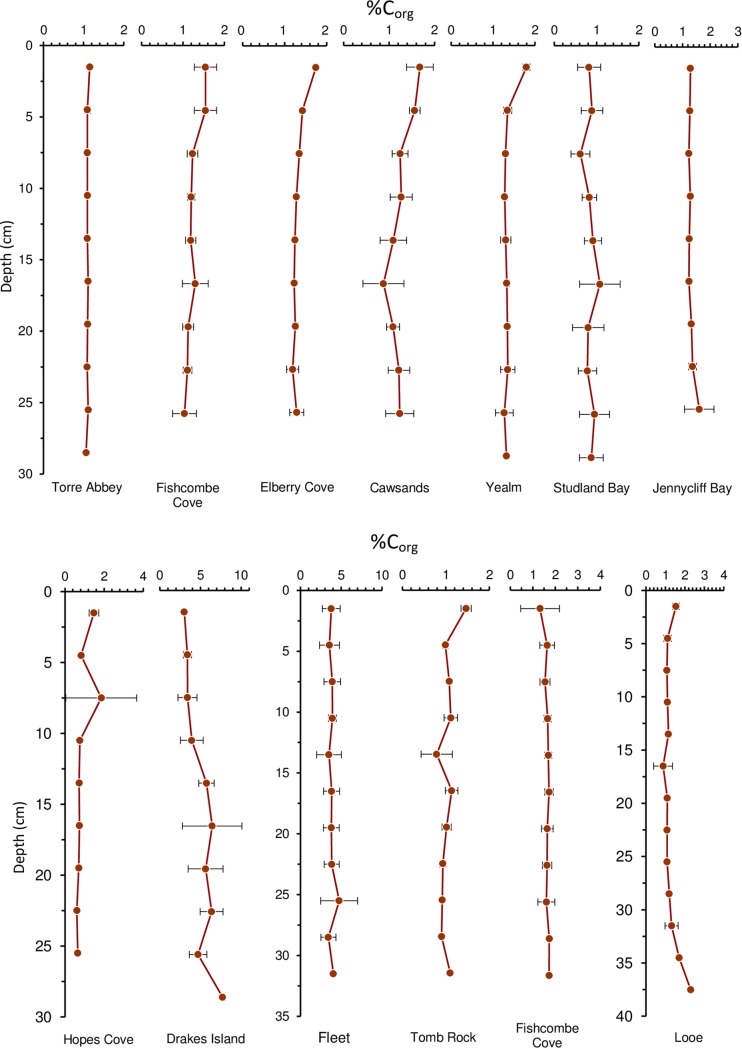
Depth profiles of the top 25-40cm of sediment cores from the average at each site. OC expressed as a percentage of the dry weight. Note the variations in x and y axis among some of the sites.

Our empirically derived relationship was not as strong as the equation derived from the global literature (R^2^ = 0.38 vs R^2^ = 0.96) [13]. To assess the reliability of the developed equation the %OM results were put through both equations and differences were statistically analysed. The differences were not significant (p<0.001), so analysis was based on our linear equation. Average %OC content ranged from 0.86 ± 0.27% (Studland Bay) to 4.94% ± 2.00% (Drakes Island). Mean %OC was 1.70% ± 1.23% and median %OC was 1.28%. Sediment profiles showed no change at depth (Fig 2). As with %OM, %OC at the Fleet (3.82% ± 1.14%) and Drakes Island (4.94% ± 2.00%) were significantly higher than all other sites (p < 0.001).

Integrated over a depth profile of 30cm the C_stock_ of UK seagrass meadows ranged from 29.40 ± 0.65 Mg C ha (Tomb Rock) to 114.02 ± 21.45 Mg C ha (Drakes Island), more than twice the value of the next highest C_stock_ (42.07 ± 3.08 Mg C ha at Cawsands), with an average of 41.54 ± 4.54 Mg C ha (Table 2). Despite the high %OC at the Fleet the low DBD meant that its C_stock_ was below average among the sites (37.76 ± 3.84 Mg C ha). Removing Drakes Island from the data reduces the range substantially with an average of 37.02 ± 4.22 Mg C ha. To allow for global comparisons C_stock_ was extrapolated to 100cm as per the IPCC guidelines for coastal wetlands [29, 31]). The 100cm depth-integrated C_stock_ among sites ranged from 98.01 ± 2.15Mg C ha (Tomb Rock) to 380.0 ± 71.51 Mg C ha (Drakes Island), with an average of 140.98 ± 73.32 Mg C ha. In both cases (C_stock_ 30cm and 100cm) there was a significant difference between the total C_stock_ of Drakes Island compared with all other sites. There were no significant differences between any other sites.

Sediment characteristics also varied between sites, ranging from sand to sandy silt. Sediment silt content ranged from 1.99% ± 0.66% (Studland Bay) to 29.92% ± 5.30% (the Fleet). Only Studland Bay and Drakes Island were statistically different from one another (p < 0.001). The Folk and Ward description of sorting [31] ranged from Moderately Well Sorted to Very Poorly Sorted among sites (Table 2). The Fleet was the least well sorted (Very Poor), Cawsands and the Yealm were also Poorly Sorted. The remaining sites were Moderately and Moderately Well sorted.

Plant density ranged from 2.84 ± 4.75 plants per 50cm^2^ (Jennycliff Bay) to 53.53 ± 10.45 plants per 50cm^2^ (Studland) (Table 2). Studland Bay had statistically higher aboveground biomass than any other site (p < 0.001), all other sites were the same. This contradicts the visual inspection of the sites and may show that taking biomass from the centre of the meadow is misrepresentative of the whole site. Many sites recorded high standard deviation compared to average plant count, highlighting the patchiness of some sites. Patchiness was particularly pronounced at Fishcombe Cove (5.71 ± 7.64 per50cm^2^) and Jennycliff Bay (2.84 ± 4.75 per50cm^2^). Standard deviation was high across all sites apart from Studland Bay (53.53 ± 10.46cm^2^), which was the most consistently dense meadow.

In general, the surveyed meadows ranged from dense uninterrupted beds (Fleet, Studland, Drakes Island) to open sand with small patches of seagrass cover (Cawsand, Firestone bay). The Fleet and Studland Bay both contain large bare patches within their dense beds, the Fleet for reasons currently unknown and Studland Bay because it is a popular anchorage and contains numerous anchor scars. Site exposure differed among sites. The Fleet is a lagoon, flanked by Chesil Bank and connected to the sea by a narrow channel to the south that leads into Portland Harbour. In comparison, the meadow at Torre Abbey lies in the middle of a large bay, ~500m from shore, with frequent through traffic from the port, and no protection from oncoming weather.

Meadow size varied from 274.68ha (the Fleet) to 0.14ha (Yealm) with most sites smaller than 60ha (Table 1). Sea depth of site ranged from 2.5m (Studland Bay) to 7.7m (Hopes Cove). Average site depth was 5.10 ± 1.60m. The environmental data showed very weak regression relationships between all parameters and C_stocks_: C_stock_ and plant density (R^2^ = 0.003); C_stock_ and average site depth (R^2^ = 0.034); C_stock_ and sediment silt content (R^2^ = 0.064); C_stock_ and size (R^2^ = 0.021); C_stock_ and dry bulk density (R^2^ = 0.012) and; C_stock_ and %OM (R^2^ = 0.372).

## Discussion

This study is the first to estimate seagrass C storage in the UK. It demonstrates that despite contrasting habitat features there is little variation in the C_stocks_ among sub-tidal *Z*. *marina* habitats, existing on the same latitude along the southwest coast of the UK. These results contradict a growing body of literature that has found variations in the C storage of seagrass meadows among habitats formed of the same species [12,13,22,24,29,31]. Although documenting large variation these studies were unable to provide an adequate understanding of factors influencing OC accumulation and storage. Our results suggest that habitat conditions do not meaningfully influence the C_stock_ within the UK’s seagrass meadows. The mechanisms which influence sediment C accumulation in seagrass meadows remain unclear.

Drakes Island appears to be exemplar in its C storage ability in the region. The 100cm depth integrated C_stock_ at Drakes Island is nearly three times higher (380.07 ± 17.51 Mg C ha) than the average of all other sites (140.98 ± 73.32 Mg C ha). All other sites contained similar C_stocks_ ranging from 98.01 ± 2.15 Mg C ha to 140.24 ± 10.27 Mg C ha. Other studies have found that accumulation of fine-grained sediments within seagrass beds significantly influences seagrass C storage [8,25,32]. The relationship between sediment silt content and C_stock_ among these sites was weak, suggesting this was not an influencing factor among sites. Drakes Island had one of the lowest sediment silt contents (5.51 ± 1.43%) and the site with the highest silt content (Fleet 29.92 ± 5.30) did not have particularly high C_stock_, although its %OC (3.82 ± 1.14%) was high and the low C_stock_ is likely due to the low dry bulk density at the site (0.34 ± 010). Aboveground biomass is also attributed to higher C_stocks_ among seagrass meadows [33], though this was not evident in the data (R^2^ = 0.003). Studland Bay had by far the highest average plant count (53.53 ± 10.45 per 50cm^2^) (Table 2), but an average C_stock_ (37.76 ± 5.39). Plant count at Drakes Island was reasonably high (10.42 ± 8.40 per 50cm^2^) but standard deviation was also high, suggesting a less uniform cover of dense growth. Patchiness within sites was generally high, indicating potentially poor ecosystem health [11]. Fishcombe Cove (5.71 ± 7.64), Jennycliff Bay (2.84 ± 4.75), Firestone Bay (4.05 ± 5.88) and Yealm (6.70 ± 7.01) all displayed vast variations among surveyed quadrats but overall no relationship was noted between plant count, or patchiness, and C_stock_.

That the expected trends are not identified within these results should not render them insignificant. It is likely that the high OC content found at the Fleet is in part attributable to the high sediment silt content. More intricate factors are likely involved that allow Drakes Island to store more C where its sediment is less suited and restrict Studland’s sequestration capacity where its canopy is more favourable. This study was unable to assess the sources of C within the seagrass meadows, which can be an important influencing factor determining C_stocks_ [25]. Sources of C contributed to up to 73% of the difference between C storage in *Z*. *marina* habitats in the Nordics [25]. On average 50% of sedimentary OC is derived from allochthonous sources [14,34], and it may be that the ratio of C contribution (*Z*. *marina*: external sources) is an influencing factor. Further analysis should be considered to understand the relationships between C_stock_, silt content and aboveground biomass among these sites.

Seagrass systems typically have very little sediment turnover [35]. C diagenesis causes a gradual breakdown of labile and later increasingly stable C [35]. The result is normally a decrease in organic matter at depth. The sediment profiles at these sites did not fit this trend. It may be that the shallow 30cm cores are not sufficiently deep to describe the expected negative exponential profile that represents OC decomposition with age [20]. However, other studies have recorded this with similar core lengths [23]. Deep cores (1-2m) at key sites should be taken to fully examine this relationship.

### C stock comparisons

**Fig 3:**
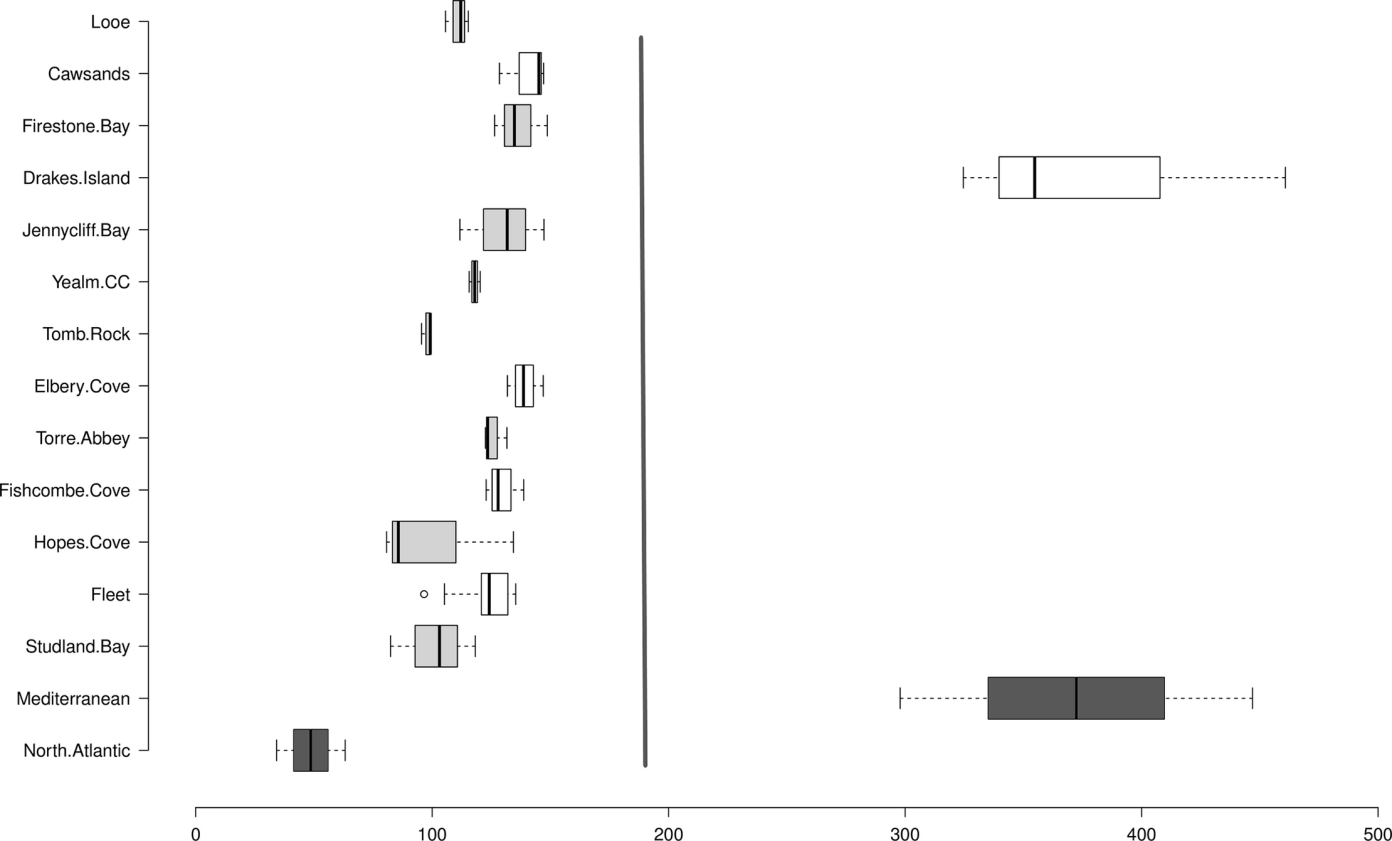
Average C_stock_ of 13 seagrass meadows along the southwest coast of the UK with regional comparisons (dark grey Mediterranean and North Atlantic) and global average (grey line) extrapolated from [12].

The mean sediment C_stock_ for the top 100cm of sediment (140 ± 73.32 Mg C ha) was just short of the global average of 194.2 ± 20.2 Mg C ha [13] (Fig 2). The range of C_stock_ between sites was large (98.01–380 Mg C ha), but greatly reduced when Drakes Island (380.07 ± 71.51 Mg C ha), was removed (98.01–140.24 Mg C ha). Four sites fell below the globally documented range of 115.5–829.2 Mg C ha (from 41 100cm cores), though when you include the global extrapolated data from cores at least 20cm deep (extrapolated to 100cm), the range widens from 9.1–829.2 Mg C ha [13]. In these cases, values tend to be lower, so deeper cores at the surveyed sites may well reveal higher C stores. All the surveyed sites contain average C_stock_ well above the average for North Atlantic seagrass meadows (48.7 ± 14.5 Mg C ha) (Fig 3) and increased the number of data points from 24 to 37 [13]. Surprisingly, Drakes Island is comparable to the Mediterranean averages, dominated by *Posidonia oceanica* (Fig 3). It is not uncommon for sites to exhibit C stores well above those within its region [25]. Understanding the mechanisms behind the C stores at Drakes Island might help to deepen our understanding of seagrass C storage.

Mediterranean cores contribute 22% of the total data for the global average, skewing the average substantially. The disparities between our results and the average for the North Atlantic further highlight the dangers of using global and regional data as a proxy for local seagrass C storage. There is a growing desire to use seagrass blue C as a mechanism to increase seagrass protection worldwide. Blue C research has come under recent scrutiny [36] and to maintain robustness we must be transparent about the services provided by local habitats, and refrain from overgeneralising. The C_stock_ values documented for the UK’s seagrass meadows fall within the upper range of those recorded in the rest of Europe. Across Europe estimates of *Z*. *marina* C_stock_ vary considerably, ranging from 500 ± 50.00 g C m^2^ to 4324.50 ± 1188.00 g C m^2^ in the top 25cm of sediment [25,37,38] (Table 3). With an average C_stock_ of 3372.47 ± 1625.79 g C m^2^ the UK is second only to Denmark. The variation between regions is considerable and both the UK and Denmark contain anomalous sites with significantly higher C_stocks_ than the rest of their region; 8649.93 ± 2330.02 (Drakes Island) and 26 138 ± 385.00 (Thurøbund) respectively, but also consistently higher C_stocks_ across all sites when compared to the rest of Europe.

As with Drakes Island no obvious explanation for the Danish sites high C content was given above its location in a ‘relatively sheltered site’ and large amounts of organic sediments [25]. This study found greater variations in the C_stocks_ of eelgrass sediments than our study noting that eelgrass production, root: shoot ratio and contribution of *Z*. *marina* to the C pool explained 67% of the variation. Similar analysis at the sites included in this study would make an interesting comparison here. The large variation among regions demonstrated by these studies further highlights the danger of using global and regional data as a proxy for estimating local blue C values. It also confirms that even within species there is considerable variation in seagrass C storage capacity and suggests that abiotic factors are more important than biological. Although the drivers remain unclear, the C stored in the seagrass meadows along the southwest coast of the UK represent one of the largest known stocks within Europe and, therefore, represent important sites for further study and conservation.

**Table 3:** Mean C_stocks_ in European *Z*. *marina* meadows in the literature and present study.

Country	Region	C_stock_ ± Stdev (g C m^2^)	Depth (cm)	No. sampling locations	Reference
Denmark	Baltic Sea, North Sea	4324.50 ± 1188.00	25	10	(25)
UK	Southwest coast, English Channel	3371.47 ± 1625.79	25	13	Present study
Sweden	Southern Sweden, Baltic Sea	2000.00 ± 2121.32	25	5	(38)
Portugal	Southern Portugal, North Atlantic	1000.00 ± 120.00	25	2	(38)
Finland	Southern Finland, Baltic Sea	627.00 ± 25.00	25	10	(25)
Bulgaria	Eastern Bulgaria, Black Sea	500.00 ± 50.00	25	2	(38)
Poland	Puck Bay, Baltic sea	148.21 ± 90.31	10	3	(39)

That seagrass meadows can also be a source of CO_2_ and atmospheric methane (CH_4_) has largely been neglected in the literature [39,40]. A recent study suggests that seagrass could be contributing up to 30% more to the global CH_4_ emissions than previously thought, and calls for these emissions to be included in seagrass C calculations [36]. There also lacks at the root of blue C science adequate understanding of how OC accumulated in soils can be remineralised to CO_2_ and re-released back into the water column, where it has the potential to enter the atmosphere [40]. A recent paper suggests the dissolution of calcium carbonate from the inorganic C pool has the potential to buffer the C sequestration capacity of seagrass meadows, in some cases perhaps shifting habitats to C sources [40]. These mechanisms need more exploration and will vary regionally. Regardless, they call into question the reliability of global seagrass C sequestration estimates. Unfortunately, these considerations are outside the scope of this study, the core aim of which to provide the first estimates of C standing stock in the UK’s seagrass sediments. We argue that this data is much needed, especially within the current climate of forwarding marine conservation goals in the UK. Thus, stock calculations alone provide vital, much needed, information on this under-studied habitat. Hopefully, future studies can investigate the flux of C, and further add to the data pool both locally and globally.

### Significance of C stocks for UK

There were marked differences in the sizes of seagrass beds in the surveyed sites, and by association, the C pools within each bed (Table 4). Total C pool in the top 100cm of the surveyed sites ranged from 14.52 t C at Tomb Rock to 33,578.31 t C at the Fleet. Despite the high C_stock_ found within Drakes Island, the site itself is very small (4.25ha) and contains only an estimated 1,616.67 t C within the top 100cm of its sediments. The estimated C pool in the top 100cm of the 13 surveyed sites along the southwest coast of the UK was 66,337 t C, or the equivalent of 10,512 individuals UK peoples CO^2^ emissions per year. This is clearly not a significant number in terms of the UK’s GHG emissions. However, for an area covering half the size of Richmond Park (London’s largest park) this figure is significant relative to its size. The Fleet is a large seagrass bed and contains 10% of the annual CO^2^ emissions of its closest town (Weymouth). The seagrass beds within this study make up a fraction of those found in the UK. A number of studies have estimated the cover of seagrass meadows in the UK, although the actual extent remains uncertain [41–44]. The total mapped area of *Z*. *marina* is 4887ha [43], though not all seagrass beds in the UK have been mapped. This figure is derived from some Special Areas of Conservation (SAC) and additional data from published studies only [44]. A reasonable estimated extent of seagrass seems to fall between this number and 10,000ha [41–44]. Taking the average from this study the estimated total standing stock of C in the UK’s seagrass meadows is, therefore, between 108,427 and 221,870 t C. This is substantially higher than the Garrard & Beaumont [37] estimates which used *Z*. *marina* C stocks from European sites, estimating that the UK’s seagrass meadows had the potential to store between 8050–16,100 t C. To fully grasp the significance of the UK’s seagrass C stocks a full inventory of the UK’s seagrass habitats should be completed and sediment cores from a wider range of meadows analysed. Further, the sequestration rate of these beds should be analysed to understand how much C per year these sites are able to sequester. Using the UK governments estimated traded central C value for 2017 of £24/t [45], the UK’s seagrass sedimentary C stock has a monetary value of between £2.6 million and £5.3 million or an average of £3,360/ha in the top 100cm.

**Table 4:** Mean C_stock_ and equivalent monetary value of seagrass meadows along the southwest coast of the UK.

Site	C_stock_ 100cm (Mg C ha)	C_stock_ 25cm (g C m^2^)	Size (ha)	Total C (Mg C ha)	Monetary value
Looe	111.00 ± 4.91	2643.48 ± 146.31	56.52	6273.74	£150,570
***Plymouth Sound***					
Cawsands	140.24 ± 10.27	3436.78 ± 228.89	11.77	1650.65	£39,616
Firestone Bay	136.62 ± 11.26	3253.35 ± 271.38	0.76	103.83	£2,492
Drakes Island	380.07 ± 17.51	8649.93 ± 2330.02	4.25	1615.28	£38,767
Jennycliff Bay	130.25 ± 17.83	3273.08 ± 95.31	11.77	191.46	£4,595
Yealm CC	117.97 ± 2.34	2882.59 ± 10.05	0.14	16.16	£388
Tomb Rock	98.01 ± 2.15	2396.87 ± 69.82	0.15	14.52	£349
***Torbay***					
Elbery Cove	139.13 ± 7.60	3343.82 ± 204.30	29.31	4077.79	£97,867
Torre Abbey	125.87 ± 5.00	2995.01 ± 119.94	104.11	13105.65	£314,536
Fishcombe Cove	129.82 ± 8.12	3175.36 ± 143.14	0.23	29.86	£717
Hopes Cove	100.26 ± 29.62	2539.40 ± 812.30	2.73	273.71	£6,569
***Weymouth/ Poole***					
Fleet	122.25 ± 12.80	2849.96 ± 376.33	274.68	33578.38	£805,881
Studland Bay	101.25 ± 18.00	2389.48 ± 432.16	53.37	5403.96	£129,695

C_stock_ Mg C ha = mean megagrams of C per hectare over 100cm profile ± standard deviation; C_stock_ g C m2 = mean grams C per M^2^ over 25cm profile ± standard deviation; Size = meadow size; total C = total C in top 100cm Mg C ha

### Conservation implications for C stocks in UK

This study adds to the growing literature base that highlights the importance of the UK’s seagrass habitats [4,11,46]. Despite the growing knowledge that *Z*. *marina* beds in the UK are important nursery grounds for economically important fish species [46], and that they are mostly in a poor ecological condition [11], conservation of these habitats is lacking.

Studland Bay is the only site without any legislative protection, though it is being considered for designation as a Marine Conservation Zone (MCZ) this year (2018). The remaining sites are protected either as Special Areas of Conservation (SACs) or as MCZs, apart from the Fleet, which is a SAC, a Site of Special Scientific Interest (SSSI), a RAMSAR site (Wetlands), a Special Protected Area (SPA) and a UNESCO world heritage site (Table 1). Despite these designations there are no restrictions on dropping anchor at any of the SAC or MCZ sites. Studland Bay, Fishcombe Cove and Cawsands are favoured anchorage sites for yachters and have several anchor scars within their meadows. The impact of anchoring activities on seagrass beds is contested, especially in the UK where the yachting community are greatly opposed to any anchorage restrictions. However, a recent paper [47] has unequivocally demonstrated that direct scouring of the bed by anchors, and the subsequent resuspension and loss of fine-grained sediments as a consequence, has resulted in a loss of OC content in disturbed areas. Scars showed evidence of intensive sediment mixing, which lead to the OC stocks being significantly lower than sediments under undisturbed seagrass [47]. In the UK, moorings, which are also present at Studland Bay, have also been shown to negatively impact seagrass cover with one mooring chain potentially responsible for the loss of up to 122m^2^ of local seagrass [48].

Studland Bay is one of the most highly contested seagrass sites in the UK, with forceful opinion on either side as to whether it should be designated as an MCZ. It provides a habitat for numerous commercially important (bass, bream, flatfish) and endangered (undulate ray) species, as well as being the only known breeding ground for both species of seahorses (*Hippocampus hippocampus* and *Hippocampus guttulatus*) found in the UK [49]. Further, it is recognised by Natural England as one of the best recovered sites since the decimation of the UK’s seagrass meadows by wasting disease in the 1920s [49].

The seagrass bed in Studland Bay is a frequented anchorage for yachters coming out of Poole Harbour, who drop anchor in their hundreds during the summer months [50]. The anchor scars are visible from satellite images and cause obvious disruption to the otherwise dense meadow. The yachting community have successfully countered 15 years’ worth of lobbying to protect this site under UK law. Initially included in the original proposal for 127 MCZ designations across England in 2011, Studland Bay was excluded from tranche one (2013) and two (2016), due mainly to the objections of local people and the yachting community [51]. It has been included for consideration in the third tranche, though it is likely to gain serious resistance from the local and yachting community. Arguably, part of the reason for their adamant resistance to marine protection or the introduction of ecologically friendly moorings has been the focus on a flagship species approach to conserving this habitat, i.e. efforts have fixated on highlighting the fact that the site is a breeding ground for two protected seahorse species [52]. The calls to protect these species have largely fallen on unsympathetic ears. The attempts have created a turbulent relationship between the conservation and yachting community so that now any efforts to approach a mutual resolution are met with animosity. The flagship species approach is one that often fails to entice the diversity of stakeholders needed to ensure effective conservation [53]. By widening the debate, to include a potentially growing C stock, a more positive dialogue may be allowed to develop. By taking a monetary approach to conserving this site there is reasonable argument in favour of protecting the C found within. The total estimated C in the top 100cm of the seagrass meadow at Studland Bay is 5,403 t which has a monetary value of £129,695. The estimated value of recreational and harbour activities that are argued to be affected by conservation management in Studland Bay totals £81,100 [49].

## Conclusions

This study provides the first data on *Zostera marina* sediment C storage in the UK and offers a more accurate estimation of seagrass blue C stocks in UK waters. The work brings 13 more seagrass meadows into the global and regional dataset and, like many other studies, highlights uncertainties surrounding the variances in sediment C storage. The results show considerable uniformity, which is unusual, and, in line with other research, indicate an incomplete understanding of the factors that influence this [13,14,23,25,32]. Considered alone, the uniformity of the sites within this study suggests abiotic factors are not a strong driver of sediment C variability. However, when estimates of C storage from other European *Z*. *marina* meadows are considered it seems clear they are the primary cause of variance. Although unable to identify the drivers for this, the seagrass meadows along the southwest coast of the UK contain C_stocks_ that are significant in a European context and are, therefore, important both ecologically and in terms of ecosystem services to the region. We would argue that, for blue C purposes at least, grouping seagrass into bioregions is not a useful way to discuss similarities or differences, as even the same species within the North Atlantic bioregion vastly contradict each other.

Studies like this provide an essential snapshot of the complex processes that influence C sequestration. Detailed analysis of sedimentary structure, hydrodynamic regime, and seagrass canopy structure is vital if we are to better understand the causes of variation. Without this detail, global estimates will remain unreliable. Only by documenting inter-habitat variability will we be able to extrapolate the importance of seagrass ecosystems in a meaningful way, and thereby justify and promote measures for their improved protection.

## References

[1] Smith SV. Marine macrophytes as a global carbon sink. Science (80-). 1981;211(4484): 838–40. 10.1126/science.211.4484.838 17740399

[2] Nellemann C, Nellemann C., Corcoran E., Duarte C. M., Valdés L., De Young C., et al Blue carbon.A rapid response assessment. United Nations Environment Programme, GRID-Arendal; 2009Available from: http://www.grida.no/publications/145

[3] Hiscock, K., Sewell, J. & Oakley, J. Marine health check 2005. A report to gauge the health of the UK ‘ s sea-life. WWF-UK. 2005; Available from: http://www.marlin.ac.uk/assets/pdf/marine_healthcheck05.pdf.

[4] Jackson EL, Rowden AA, Attrill MJ, Bossey SJ, Jones MB, Jackson EL. The importance of seagrass beds as a habitat for fishery species. An Annu Rev. 2001;39: 269–303.

[5] Fonseca MS, Cahalan JA. A preliminary evaluation of wave attenuation by four species of seagrass. Estuar Coast Shelf Sci. 1992;35(6): 565–76.

[6] Hemminga MA, Duarte CM. Seagrass ecology Cambridge University Press; 2000. 298 p.

[7] Orth RJ, Carruthers TJB, Dennison WC, Duarte CM, Fourqurean JW, Heck KL, et al A Global Crisis for Seagrass Ecosystems. Bioscience. 2006;56(12): 987–96.

[8] Kennedy H, Beggins J, Duarte CM, Fourqurean JW, Holmer M, Marbá N, et al Seagrass sediments as a global carbon sink: Isotopic constraints. Global Biogeochem Cycles. 2010;24(4): 10.1029/2010GB003848

[9] Short F.T., Carruthers T.J.R., Waycott M., Kendrick G.A., Fourqurean J.W., Callabine A., et al Zostera marina. The IUCN red list of threatened species. IUCN Global Species Programme Red List Unit. 2010 Available from: http://www.iucnredlist.org/details/153538/0.

[10] Davison DM, Hughes DJ. Zostera biotopes. An overview of dynamics and sensitivity characteristics for conservation management of marine SACs. Scottish Association for Marine Science (UK Marine SACs Project). 1998 Available from: http://www.ukmpas.org/pdf/Detailed_Marine_Communities_Reports/zostera.pdf.

[11] Jones BL, Unsworth RKF. The perilous state of seagrass in the British Isles. R Soc open Sci. 2016;3(1): 150596 A 10.1098/rsos.150596 26909188 PMC4736943

[12] Foden J, Brazier P, Best M, Scanlan C & Wells E. Water Framework Directive Development of Classification Tools for Ecological Assessment: Intertidal Seagrass (Marine Angiosperms). 2010. Available from: http://www.wfduk.org/search/angiosperm.

[13] Fourqurean JW, Duarte CM, Kennedy H, Marbà N, Holmer M, Mateo MA, et al Seagrass ecosystems as a globally significant carbon stock. Nat Geosci. 2012;5(7): 505–9.

[14] Duarte CM, Kennedy H, Marbà N, Hendriks I. Assessing the capacity of seagrass meadows for carbon burial: Current limitations and future strategies. Ocean Coast Manag. 2013;83: 32–38.

[15] McLeod E, Chmura GL, Bouillon S, Salm R, Bjork M, Duarte CM, et al A blueprint for blue carbon: Toward an improved understanding of the role of vegetated coastal habitats in sequestering CO2. Frontiers in Ecology and the Environment. 2011; 9: 552–560.

[16] Pendleton L, Donato DC, Murray BC, Crooks S, Jenkins WA, Sifleet S, et al Estimating Global “Blue Carbon” Emissions from Conversion and Degradation of Vegetated Coastal Ecosystems. PLoS One. 2012; 7(9): 10.1371/journal.pone.0043542 22962585 PMC3433453

[17] Duarte CM, Losada IJ, Hendriks IE, Mazarrasa I, Marbà N. The role of coastal plant communities for climate change mitigation and adaptation. Nat Cli Cha. 2013;3; 961–968.

[18] Laffoley D & Grimsditch G. (eds). 2009 The management of natural coastal carbon sinks IUCN, Gland, Switzerland. 53 pp.

[19] Mateo MA, Romero J, Pérez M, Littler MM, Littler DS. Dynamics of Millenary Organic Deposits Resulting from the Growth of the Mediterranean Seagrass Posidonia oceanica. Estuar Coast Shelf Sci. 1997;44(1): 103–10.

[20] Serrano O, Mateo MA, Renom P, Julià R. Characterization of soils beneath a Posidonia oceanica meadow. Geoderma. 2012 S;185–186: 26–36.

[21] Hendriks I, Sintes T, Bouma T, Duarte C. Experimental assessment and modelling evaluation of the effects of the seagrass Posidonia oceanica on flow and particle trapping. Mar Ecol Prog Ser. 2008;356: 163–73.

[22] Kerr J. Introduction to Energy and Climate. CRC Press; 2017. 470 p.

[23] Lavery PS, Mateo M-N, Serrano O, Rozaimi M. Variability in the Carbon Storage of Seagrass Habitats and Its Implications for Global Estimates of Blue Carbon Ecosystem Service. PLoS One. 2013;8(9). 10.1371/journal.pone.0073748 24040052 PMC3764034

[24] Mtwana Nordlund L, Koch EW, Barbier EB, Creed JC. Seagrass ecosystem services and their variability across genera and geographical regions. PLoS One. 2016;11(10). 10.1371/journal.pone.0163091 27732600 PMC5061329

[25] Röhr ME, Boström C, Canal-Vergés P, Holmer M. Blue carbon stocks in Baltic Sea eelgrass (Zostera marina) meadows. Biogeosciences. 2016;13(22): 6139–53.

[26] Bell-James J. Developing a Framework for Blue Carbon in Australia: Legal and Policy Considerations. Unswlj [Internet]. 2016;(2011): 1583–611.

[27] MMO. ENVIRONMENTAL PROTECTION LICENSING (MARINE) MARINE POLLUTION. The Marine Licensing (Exempted Activities) Order 2011. 2011 Available from: http://www.legislation.gov.uk/uksi/2011/409/pdfs/uksi_20110409_en.pdf

[28] Walter E. Dean WE Jr. Determination of Carbonate and Organic Matter in Calcareous Sediments and Sedimentary Rocks by Loss on Ignition: Comparison With Other Methods. SEPM J Sediment Res. 1974;44(1): 242–8.

[29] Santisteban J.I., Mediavilla R., López-Pamo E. et al Journal of Paleolimnology (2004) 32: 287 10.1023/B:JOPL.0000042999.30131.5b

[30] Howard J, Hoyt S, Isensee K, Pidgeon E, Telszewski M. Coastal Blue Carbon: Methods for Assessing Carbon Stocks and Emissions Factors in Mangroves, Tidal Salt Marshes, and Seagrass Meadows. Conserv Int Intergov Oceanogr Comm UNESCO, Int Union Conserv Nature Arlington, Virginia, USA. 2014. Available from: thebluecarboninitiative.org.

[31] Blott SJ, Pye K. GRADISTAT: a grain size distribution and statistics package for the analysis of unconsolidated sediments. Earth Surf Process Landforms. 2001;26(11): 1237–48.

[32] Miyajima T, Hori M, Hamaguchi M, Shimabukuro H, Adachi H, Yamano H, et al Geographic variability in organic carbon stock and accumulation rate in sediments of East and Southeast Asian seagrass meadows. Global Biogeochem Cycles. 2015;29(4):397–415.

[33] Samper-Villarreal J, Lovelock CE, Saunders MI, Roelfsema C, Mumby PJ. Organic carbon in seagrass sediments is influenced by seagrass canopy complexity, turbidity, wave height, and water depth. Limnol Oceanogr. 2016;61(3). 10.1002/lno.10241

[34] Kennedy H, Beggins J, Duarte CM, Fourqurean JW, Holmer M, Marbá N, et al Seagrass sediments as a global carbon sink: Isotopic constraints. Global Biogeochem Cycles. 2010;24(4). 10.1029/2010GB003848

[35] Burdige DJ. Preservation of Organic Matter in Marine Sediments: Controls, Mechanisms, and an Imbalance in Sediment Organic Carbon Budgets? Chem. Rev. 2007;107(2): 467–485. 10.1021/cr050347q 17249736

[36] Johannessen SC, Macdonald RW. Geoengineering with seagrasses: is credit due where credit is given? Environ Res Lett. 2016;11(11): 113001 10.1088/1748-9326/11/11/113001

[37] Dahl M, Deyanova D, Gütschow S, Asplund ME, Lyimo LD, Karamfilov V, et al Sediment Properties as Important Predictors of Carbon Storage in Zostera marina Meadows: A Comparison of Four European Areas. Wang X, editor. PLoS One. 2016;11(12): e0167493 10.1371/journal.pone.0167493 PMC5147920 27936111

[38] Jankowska E, Michel LN, Zaborska A, Włodarska-Kowalczuk M. Sediment carbon sink in low-density temperate eelgrass meadows (Baltic Sea). J Geophys Res Biogeosciences. 2016;121(12): 2918–34.

[39] Garcias-Bonet N, Duarte CM. Methane Production by Seagrass Ecosystems in the Red Sea. Front Mar Sci. 2017;4:340 10.3389/fmars.2017.00340

[40] Howard JL, Creed JC, Aguiar MVP, Fourqurean JW. CO 2 released by carbonate sediment production in some coastal areas may offset the benefits of seagrass “Blue Carbon” storage. Limnol Oceanogr. 2018;63(1): 160–72.

[41] Davidson DM, Hughes DJ. Zostera Biotopes (volume I). An overview of dynamics and sensitivity characteristics for conservation management of marine SACs. 1998;1(95). Available from: http://www.ukmarinesac.org.uk/pdfs/zostera.pdf.

[42] Maddock A. UK Biodiversity Action Plan Priority Habitat Descriptions. UK Biodivers Action Plan; Prior Habitat Descr BRIG (ed Ant Maddock). 2008; Available from: http://www.jncc.gov.uk/page-5155.

[43] Luisetti T, Jackson EL, Turner RK. Valuing the European 'coastal blue carbon' storage benefit. 2013; 71(1–2):101–6. 10.1016/j.marpolbul.2013.03.029.45 23623654

[44] Garrard SL, Beaumont NJ. The effect of ocean acidification on carbon storage and sequestration in seagrass beds; a global and UK context. Mar Pollut Bull. 2014;86(1–2): 138–146 10.1016/j.marpolbul.2014.07.032 25103900

[45] Decc. A brief guide to the carbon valuation methodology for UK policy appraisal. 2011. Available from: https://www.gov.uk/government/publications/carbon-valuation-methodology-for-uk-policy-appraisal.

[46] Bertelli CM, Unsworth RKF. Protecting the hand that feeds us: Seagrass (Zostera marina) serves as commercial juvenile fish habitat. Mar Pollut Bull. 2014;83(2): 425–9. 10.1016/j.marpolbul.2013.08.011 23998854

[47] Serrano O, Ruhon R, Lavery PS, Kendrick GA, Hickey S, Masqué P, et al Impact of mooring activities on carbon stocks in seagrass meadows. Sci Rep. 2016;6 Available from: https://www.nature.com/articles/srep23193.pdf. 10.1038/srep23193 PMC4793266 26979407

[48] Unsworth RKF, Williams B, Jones BL, Cullen-Unsworth LC. Rocking the Boat: Damage to Eelgrass by Swinging Boat Moorings. Front Plant Sci. 2017;8:1309 10.3389/fpls.2017.01309 28791040 PMC5526064

[49] DEFRA. Department for Environment, Food and Rural Affairs Consultation on Sites Proposed for Designation in the third tranche of Marine Conservation Zones. 2018. Available from: www.gov.uk/government/publications.

[50] Collins KJ, Suonpää AM, Mallinson JJ. The impacts of anchoring and mooring in seagrass, Studland Bay, Dorset, UK. Underw Technol. 2010;29(3): 117–123.

[51] Defra. Consultation on proposals for the second tranche of Marine Conservation Zones: Summary of responses. 2016; Available from: www.defra.gov.uk%0Awww.gov.uk/defra.

[52] Garrick-Maidment N, Trewhella S, Hatcher J, Collins KJ, Mallinson JJ. Seahorse Tagging Project, Studland Bay, Dorset, UK. Mar Biodivers Rec. 2010: 10.1017/S175526721000062X

[53] Simberloff D. Flagships, umbrellas, and keystones: Is single-species management passe in the landscape era? Biol Conserv. 1998;83(3): 247–57.

